# Some Conditions of Mutual Interest to the Dentist and the Oculist

**Published:** 1891-03

**Authors:** George T. Stevens

**Affiliations:** New York


### International Dental Journal.

Vol. XII. March, 1891. No. 3.

### Original Communications.[1]

### SOME CONDITIONS OF MUTUAL INTEREST TO THE DENTIST AND THE OCULIST.[2]

BY GEORGE T. STEVENS, M.D., NEW YORK.

Among the gratifying evidences of substantial advance in the training required for the practice of medicine is the fact that a more extended and general acquaintance with the whole realm of medical knowledge is now demanded of practitioners in certain lines of special practice than was formerly exacted.

It is within the recollection of all of us that the practice of certain branches of surgery was, even quite within recent times, regarded as so far relating exclusively to the part or organ treated, that beyond the special knowledge of the mechanism of the part and the procedures usually adopted, no training in general professional knowledge was regarded as essential or desirable.

One by one these special branches have become blended more and more completely into the general practice of medicine and surgery with great advantage, both to the specialty and to the general science.

It is not many years since the optician or the jeweller performed

[1] The editor and publishers are not responsible for the views of authors of papers published in this department, nor for any claim to novelty, or otherwise, that may be made by them. No papers will be received for this department that have appeared in any other journal published in the country.

[2] Read before the New York Odontological Society, January 13, 1891.

services which are to-day regarded as among the duties demanding a high degree of technical skill and professional judgment on the part of the accomplished oculist.

Perhaps even a greater isolation from the general practice of medicine than in most other directions has existed in the practice of dentistry. The supposed local character of the defects and anomalies of the teeth, and the peculiar mechanical processes demanded in their management, led to a specializing in practice beyond that in most other branches. Fortunately, specialties in medical science are becoming less and less the field for narrow exclusions, and are more and more grounded upon a broad basis of general medical knowledge.

Without asserting that the specialist has yet reached the position, it is a pretty well recognized proposition that he should be one who, with an equipment of a broad and liberal training in general medical and surgical science, has brought an unusual amount of investigation and experience to a single department. Less and less will the practitioners in special departments regard themselves as isolated from those in other departments as the truth becomes more and more accentuated that the human organism is a unit and not merely an aggregation of parts. To attempt the discussion of the diseases of one class of organs, or the treatment of those diseases, without due consideration of all the organs of the human body, must prove, as it has always proved, in large measure, unsatisfactory. It might, doubtless, at first thought, appear, even conceding in theory what has just been advanced, that there can be little in common between the dentist and the oculist. Two special lines of surgical practice could hardly be more widely removed from each other in methods of examination or in details of procedure. I am sure, however, that a mutual understanding on the part of each of these two classes of practitioners of the conditions and methods which interest the other would contribute to the success of both.

It does not follow that the dentist should become expert with the ophthalmoscope, nor that the oculist should supply himself with the armament of the dentist. Each should, however, be thoroughly conversant with the general principles which underlie the practice of the other.

It is my purpose this evening to illustrate the mutual interdependence of these two apparently widely-separated specialties, by calling attention very briefly, not to all, but to a very few, of the conditions which should command the interest and attention of both classes.

We will glance, first, at the influence of the teeth in inducing reflex disturbances to the sight or to the functions of adjustment, and of nutrition of the eyes. Then we may consider the influence of the eyes in inducing, about the face and jaws, neuralgic disturbances, which are often erroneously attributed to the teeth. Next we may briefly refer to the influence of the eyes in the disturbance of the teeth themselves and of the jaws, and, lastly, our attention will be directed to the relations between the character of the dentist's work and the condition of his own eyes.

First of all, then, we are to examine the question of disturbances to the eyes and their functions, which may arise from the teeth. Naturally, this part of the discussion might be thought of more interest to the oculist than to the dentist; but there can be no hard and fast line of division between the interests of two classes of surgeons who must deal with conditions which are reciprocally related. The subject is not altogether new, and in this connection I need do little more than call your attention to that which is presented in the literature of your own specialty. I find in the "American System of Dentistry,"[1] edited by Litch, many pages devoted to ocular disorders induced by pathological conditions of the teeth.

The writer, Dr. Brubaker, sets out with the statement that, at the time of writing, the proposition that such disorders could be the result of reflex irritation from the teeth was hardly entertained by ophthalmologists, and he cites the absence of any allusions to the subject by Stelwag or by Wells, and to the only passing notice of any such relation by Graefe and Saemisch, as confirmation of his statement. That there was and is little in the text-books of ophthalmology upon this subject is true, and that much less than should exist is found in the periodical literature is also true. Nevertheless, I am of the opinion that ophthalmologists have in their practice recognized the relations between dental irritations and eye disorders to a greater extent than would be supposed from a cursory search of the literature. Indeed, so familiar to me was the thought of this relation, that I was quite surprised, on making such a search, at discovering that so little was actually to be found. I might refer to a number of my dental friends in confirmation of the statement that for many years I have been accustomed to send for their examination a considerable number of my patients, in whom the causes of asthenopic and other ocular symptoms have been obscure. I doubt not that many oculists have taken the same course while

[1] Vol. iii. p. 450.

neglecting to report their cases. However this may be, attention has been more especially called to the subject within the last few years than previously, and many cases are now on record which leave no doubt of the relation between dental irritation and ocular affections.

Of the large number of cases which Brubaker adduces, are some which are quite startling, and, indeed, some which must be accepted with certain reservations. For instance, a case of amaurosis of twelve years' duration is reported as cured within a few days, as the result of extracting a tooth. Now, while we may admit that a functional disturbance or even an organic disease of the eyes might be set up by an offending tooth, it would scarcely be supposed that an actual amaurosis could exist during so many years, quite independent of structural changes in the eye itself, if resulting from irritation set up by a distant organ; or, if such structural changes should have actually existed, we might reasonably expect that a cure would require a time in excess of a few days. Possibly in a case of spasm of accommodation, a condition which might easily be induced by reflex irritation, the patient might represent the confusion arising from the inability to obtain a focal adjustment as an inability to see. Something like this, indeed, often happens in the practice of the oculist. I am not infrequently informed by patients that the application of atropine to the eye, either by others or by myself, has been followed by complete blindness. In other words, the inability to see clearly an object at a given distance is in the mind of such persons associated with the idea of not seeing at all.

I suspect that a number of the long-standing cases of amaurosis, cited as being suddenly cured, may belong to this category. Nevertheless, the fact remains that whereas the eye of the patient rendered him little practical service, it was restored to usefulness after the removal of an irritating cause situated in the mouth.

Naturally, we place greater reliance upon reports of this kind when coming from a source which we can recognize as especially worthy of respect and consideration. Perhaps among those who have added considerable contributions to this subject, Galezowski may be regarded as among those least liable to be misled and best able to judge of the actual conditions represented as existing. Galezowski[1] calls attention to a variety of clinical experiences

---

[1] *Revue générale d'Ophthalmologie*, October, 1888. *Vide*, also, Widmark, *Annales d'Oculistique*, September and October, 1888. Also, Riva, in same journal.

bearing upon this subject, which he thinks has received less attention than its importance merits. According to him, during the first dentition many rebellious cases of corneal disease arise from the reflex irritation induced by difficult eruption of the teeth. Such cases recover only when the eruption of the teeth is facilitated. Neuralgic affections about the orbits, he believes, often arise from the second dentition, and finally, with the appearance of the wisdom teeth, occur still other forms of ocular troubles. Dental caries comes in for its share of ocular disturbances, one of its most common manifestations being accommodative asthenopia. This affection, he tells us, is often induced in cases where refractive errors do not exist, and he relates a number of cases of interest to illustrate his proposition. He also relates a case of blindness of one eye with no lesion shown by the ophthalmoscope, which he thinks arose from the extraction of a tooth.

It would be quite possible to put interpretations upon some of these facts somewhat different from those which are placed upon them by the distinguished oculist, while yet fully conceding the great importance of the source of irritation which he discusses. I am much disposed to regard the dental irritation existing in a considerable proportion of the cases to which reference has been made in the light of a collateral source of disturbance, rather than as the original and only disturbing element. For instance, let us take corneal ulcers, of which Galezowski speaks at greater length, perhaps, than of other eye-diseases arising from dental irritation. I observed many years since and reported the results of the observations to the International Medical Congress held in Philadelphia in 1876, facts showing that a very important factor in the causation of corneal ulcers is to be found in the anomalies of refraction of the eyes themselves.[1] My experience in later years has confirmed this view, but has led me also to regard the anomalies of the rotating muscles of the eyes as equally influential. The experience of others has in certain respects confirmed my own observation. In 1883 appeared in *Annales d'Oculistique* an article by Dr. Georges Martin, in which the writer presented facts and arguments in support of the theory that astigmatism, one of those refractive anomalies, is an extremely important factor in the etiology of corneal ulcers.[2] Other contributions in this line have added weight to this view of

---

[1] *Vide Transactions of the International Medical Congress*, Philadelphia, 1876.

[2] Sur le rapport qui existe entre une variété de la kératite grave "dite scrofuleuse" et l'astigmatisme de la cornée, *Annales d'Oculistique*, 1883, p. 15.

the subject. These writers, so far as I have observed, have taken a less extended view of this class of disturbing influences than was held by myself,—they regarding astigmatism only among these anomalies as a source of evil in connection with corneal troubles; while according to my own view, as stated in 1876, all of the anomalies of refraction were regarded as possible disturbing causes in this connection, and to which I would now add the anomalies also of the muscular apparatus.

Now, it is in this connection that I would call your attention to the very frequent disturbances in the function of development and nutrition of the teeth, which we find in children who have marked anomalies of the refractive and muscular states of the eye. This important consideration, although properly belonging to another division of this discussion, demands also a place in this connection. The excessive nervous expenditure to which children with anomalies of the ocular apparatus are subjected from the time that they begin to adjust the eyes may well be supposed to react upon their physical development and upon the phenomena of physiological functions.

At the period of dentition, when unusual nervous energy is demanded, the entire supply is already in requisition for the ordinary purposes of the child, and in overcoming the difficulties of ocular adjustments, for these make great demands upon the child's nervous power. Hence the eruption of teeth is difficult, not necessarily because of unusual resistance of the tissues overlying them, but because of the inadequate nervous force to push them out and heal the wound. The irritation of this arrested process is now felt in addition to that of the ocular irritation, and the reaction, which sometimes occurs elsewhere, is in certain cases located in the eye itself. Now, relief may be had by removing either the primary exhausting or the resulting irritating cause. In either case, after the removal of one, be it the primary or the secondary cause, there is often a nervous reserve sufficient to heal the local disorder. Hence, it may happen that corneal ulcers or other ocular diseases which occur at the period of dentition may be relieved by attention to this process, while the actual predisposing or underlying cause may be found in natural defects of the refractive or muscular apparatus of the eye.

In fact, my own experience has shown, in many instances, that if, in cases of this character, we can relieve the tension upon the accommodation, or that upon the muscular apparatus of the eyes, the powers of nutrition rally, and both the corneal disease and the arrested or difficult eruption of the teeth take care of themselves.

Thus, it often happens that in a child with simple hypermetropia the application of atropine, which arrests for the time the overtaxed function of accommodation, will afford such a rest that the child at once recovers from his ulcer of the cornea and his entire physical force appears to be renewed. On the other hand, the child who is nagged by an astigmatism of not a high degree, will find equal relief if we instil into the eye a solution of eserine, or some other agent of its class, which stimulates the fretted and discouraged muscle of accommodation.

This important difference in the application of these two notable remedies has never, so far as I have observed, received any public recognition. Yet the principle has proved in my own practice a most valuable guide in the choice of an instrumentality for the relief of corneal ulcers in children.

Taking the view, then, that we have in many of the cases of corneal troubles, such as M. Galezowski has discussed, an original physical defect or group of defects which continues as a source of exhaustion and of irritation until neutralized or removed, and which may combine with dental or other local irritation and thus induce disease, we have a most important suggestion for both the oculist and the dentist.

By all means, then, let us remove the peripheral irritation, as we may find it located in the jaws; but let us not overlook a predisposing natural defect, which is very often behind this dental trouble and may give origin to it.

In what I have said upon this subject, I speak not simply from a theoretical stand-point, but from that of experience, and of extended and, as I believe, careful observation.

The second line of thought suggested in this connection is the influence of the eyes in inducing neuralgic disturbances about the face and jaws, which are often incorrectly attributed to dental irritation.

The precepts laid down by Neucourt, as I find them quoted in the "American System of Dentistry,"[1] in respect to these affections are very emphatic, and in my view might lead to erroneous practice. He says, speaking of facial neuralgia,—I quote from Dr. Brubaker's valuable article,—"When a tooth itself is the seat of pain and the patient definitely specifies it as such, there can be no doubt

---

[1] Vol. iii. p. 477.

that it is the origin of the disorder." Neucourt announces several distinct propositions relating to the causative effect of dental irritation in inducing neuralgia in the branches of the trigeminal nerves, all as positive and as little modified by exceptions as that which I have just quoted.

Admitting, as I do, and as every careful clinical observer must do, an intimate relation between the condition of the teeth and the irritations of surrounding and even of distant nerves, and recognizing clearly the fact that there may be, and sometimes is, facial neuralgia of uncomplicated dental origin, I wish to call your attention in the most emphatic manner to the errors which might result from following too closely the rules contained in the series of propositions, one of which is above quoted.

It is one of my familiar experiences to see patients who have submitted to the extraction of sound teeth, sometimes of a single tooth, sometimes of several, and in not a few instances of the whole group of teeth from one or both maxillæ, on account of neuralgia in the face or jaw, without experiencing any relief and indeed with the result of intensifying the painful disorder. Permit me to emphasize the statement by repeating it, that such experiences are with me not only not rare, but unfortunately somewhat common. In a paper on the subject of neuralgia, contributed by myself to the *Medical Record*, in 1877, I presented the details of a case of this class which is interesting as being, so far as I am aware, one in the first group of cases reported in which typical cases of neuralgia had been relieved as the result of corrections of ocular defects. In the case now referred to, the patient had suffered from pain of the most intense character, located in the face and jaw, during many years. She had been advised to submit to the removal of the teeth, all of which were sound, not only by her friends, but by a competent dentist. She had even, at the time when I saw her first, an appointment with a dental surgeon to have the supposed offending members removed. The patient was found to have a high degree of astigmatism. Glasses were given for its correction; improvement quickly followed and the entire relief which was gained continued during my acquaintance with her, which was more than a year. This case, except in the fortunate escape from the loss of the teeth, is a fair example of a very large class.

I cannot resist the temptation in this connection to submit my own personal grievance in the matter of extracting teeth for neuralgic affections of supposed dental origin. The worst of the grievance is that I am forced te acknowledge not only the error of my dentist, an able and accomplished gentleman, who was fully sustained in his practice by recognized authorities, but my own weakness in permitting what, in an hour of less overwhelming pain, I would have forbidden either in my own case, or in the case of any one of my patients.

In addition to my professional labors, which were very severe at the time of which I speak, I had undertaken other work which demanded the most severe use of the eyes during several weeks until very late every night. Suddenly I was attacked with a most violent pain in one side of my face, the most intense focus appearing to be at a particular point in the upper jaw. After a good deal of intense suffering, I consulted my dentist, who assured me that there was an abscess at the root of the second molar and that I would get relief only from the extraction of the tooth. I preferred to suffer. Again and again I went, and as the pain became daily more unbearable and my own mental condition more desperate, I yielded to the repeated advice and submitted to the removal of a perfectly sound molar, which concealed no abscess. I returned to my room, took to my bed and remained a week under the influence of hypodermic injections of morphine. Gradually I became able to resume my work, and quickly learned that any severe employment of the eyes would induce a return of pain. I was forced to relinquish the undertaking which I had commenced and permit the eyes to rest during several months. Even now, after eleven years, any unusual severity in the application of the eyes continued for a few days brings back the old pain in the very spot from which the tooth was extracted. We are told by many writers that time is not to be lost in extracting these painful teeth; if there is special tenderness on tapping, or, if the tooth protrudes so that the tooth of the opposite jaw strikes it before its fellows, the indication is decisive of the presence of an abscess. I cannot regard these signs, singly or together, as constituting undoubted evidence of abscess, and I am sure that only the most positive indications should ever warrant the sacrifice of a sound tooth, or one which the skill of the dentist can make as good as sound. Even conceding the presence of an abscess, I am aware that dental science is too far advanced to demand the sacrifice of a sound tooth, for there are methods other than extraction for the relief of an abscess.[1]

---

[1] During the discussion on this paper one of the gentlemen suggested that the practice of which I have spoken above is now obsolete. While, as I have stated, more conservative methods are known and followed, the practice to

It is not a part of my plan to discuss the diagnosis of abscess of the maxillæ. I wish only to impress the fact which is very frequently brought to my own notice, that ocular irritation is responsible for a great number of the neuralgic affections of the face, and that the wanton sacrifice of the teeth, in a blind hope of relieving pain in their vicinity, appears not to be good dentistry. I do not wish to be understood that I would permit an alveolar abscess to remain locked in until it could work its way out by some distant channel. On the other hand, I would remind the dental surgeon that he is the guardian and not the destroyer of the teeth. He should therefore satisfy himself, first, that an abscess actually exists, and, second, that he can find for it no outlet less damaging than that made by the destruction of an important organ; and, what may be of equal importance in preventing a painful affection from becoming an inflammatory one, he should be on his guard for sources of reflex disturbances, such as may be found in other parts, and especially in the eyes.

We turn now to speak very briefly of one of the affections of the jaw, which causes much trouble to the dentist, and much mental and often physical suffering to the patient. I refer to that deformity of the upper jaw called the wedge-shaped jaw. It has for a good while attracted my attention and my interest; it is that condition in which the upper jaw becomes compressed laterally and becomes angular in front, causing the upper anterior teeth to project and causing a difficulty, and in some extreme cases an impossibility, of closing the lips. Even when making a moderate effort to bring the lips in contact, a line of white shows between them, and in certain cases the whole of the front superior teeth.

This is not only a distressing defect, but one which it is to be hoped may some time be more than it is at present under the control of the dentist or physician. In all the cases which have come under my own observation, the defect has been progressive. It has not in any instance been a congenital defect, nor has it in any of

---

which I have alluded is still largely prevalent. An article in a very recent number of a prominent medical journal insists upon the early extraction of the tooth supposed to conceal an abscess. On the day following that on which this paper was read there came to my office a lady suffering from glaucoma. In this disease the eye becomes hard, and neuralgic pains of the face often constitute an important element in the symptoms. On the day before, the lady had had two sound teeth drawn in the hope of relieving the pain in the face. Thus, on the very day that our friend declared the practice obsolete, another dentist was extracting teeth for the cure of glaucoma.

those which I have studied been observed until the patient was a few years of age. In this respect, all the cases which I have noticed have followed a course somewhat similar to that of myopia. In myopia, according to the prevailing view, the subject is very rarely at birth, or in very early infancy, myopic. As the years advance, the eyeball gradually elongates, causing the patient to be near-sighted, and in most cases progressively so, the anomaly usually increasing during several years. In such progressively advancing defects, it is proper to inquire whether there is not some predisposing cause for the mal-nutrition which results in the defect, and if such cause may not be discovered and removed. So far as my observation has extended, I have found this wedge-shaped jaw only in persons who were the subjects of very marked defects in the equilibrium of the eye-muscles. This muscular condition has in all these cases induced important nervous disturbances, and I think there is ground for believing that, at least in a certain proportion of such cases, the mal-nutrition which has resulted in the defective form of the jaw is one of the reactions from the ocular disturbances. It may well be objected that the deficiency in muscular equilibrium is, like the defect of the maxillæ, acquired and progressive.

Time will not permit me to go into a discussion of this objection on this occasion, and I shall content myself by saying that the objection is not well founded; that the imperfect equilibrium of which I speak is congenital and not acquired. It is certainly the case that these ocular anomalies cause important nervous disturbances, and there is no physiological reason why a disturbing cause should not induce structural, as well as functional, irregularities. In this statement I find myself in accord with some of your most distinguished authorities.[1]

I have called attention to this defect of the jaw and its possible relation to anomalies of the ocular muscles, as a single example of a class of defects which come under the observation of the dentist, and which should lead him to the study of the nervous influences which are predisposing to them.

It is at least legitimate, when a fault of nutrition of this character is found associated with a known cause of irritation, to remove

---

[1] For instance, I find in Kingsley's well-known work on "Oral Deformities" the view, that the development of the jaws and teeth is largely governed or modified by the condition of the nervous system, frequently emphasized; and he declares that abnormal dental development and nervous diseases are correlated and spring from the same cause (page 22 to 25).

such cause as one of the steps in arresting the progress of the defect.

Let us now briefly consider the branch of our subject which has a personal interest to the dentist, namely, the influence of his special work upon his own eyes.

There are few classes of workers whose eyes are forced to perform more laborious service, and, I suspect, few in which the results are more troublesome. If we were to take a census of the strong men between the ages of thirty and forty years in the general practice of medicine and in dental practice, who suffer from oft-recurring sick headaches, from dizziness, from habitual pains about the back of the head, from frontal headache, and from various other neuroses, I feel confident that by far the greater proportion would be found not among the general practitioners, but among the dentists. When we consider the character of the dentist's work, especially of one who devotes much of his time to filling of teeth, we cannot fail to see that his eyes are harnessed to his work in an unusual manner. Hour after hour he is gazing with careful attention upon a small fixed point situated in a locality never very strongly, and often very poorly, illuminated. He cannot thus fix his gaze for a long time without making unusual demands upon the nervous energy by which the adjustments of the accommodation and of the mutual relation of the eyes is maintained. Hence, this department of dentistry must of necessity be one in which large demands upon the nervous energy of the practitioner are made, even in case of one in whom the functions of accommodation and of adjustments of the eyes are most favorable. This being the case, how much more expensive must these prolonged efforts at fixation of the eyes become to one in whom there are defects in the refractive or muscular apparatus of the eyes? If, in the first case, work is laborious, in the second it must be exhausting.

For many years I have called attention to the reflex troubles arising from difficulties in the management of the eyes, and I need not repeat what has already been said. Let me only remind you that the needless expenditure of nervous energy is a waste. One or two illustrations of such a wasteful expenditure may help to impress my meaning.

Let us suppose a person who is hypermetropic and whose occupation demands long-continued efforts at accommodation of the eyes at near points. In hypermetropia the eye is short, and a greater effort is demanded in adjusting in accommodation than in the ideal eye. Just in proportion as the hypermetropia is greater, is the demand upon the nervous energy greater in accommodation. Given, then, two persons, in other respects equal, one of whom is hypermetropic, the other not; the first in a given number of hours of labor at accommodation must expend a greater amount of energy than the second. In other words, the hypermetrop goes to his work handicapped. Again, suppose one performing a given class of work to have defective muscular arrangements of the eyes by which a greater demand than is required in equilibrium is necessary in bringing the visual lines to fix upon a given near point. It seems hardly necessary to say that the extra energy demanded in bringing these lines to the proper point and holding them there, is so much additional labor, and that of two persons otherwise equal, the one possessing the defect is at a serious disadvantage.

Not only are those persons subject to natural defects of the adjusting apparatus of the eyes at a theoretical disadvantage, but in fact, as a class, such persons are more subject to complaints arising from an exhaustion of nervous forces than others.

The natural and logical conclusion from this statement is that a class of workers who make unusually severe demands upon the eyes should make intelligent and reasonable provision that the organs from which so much is required should be placed in the best possible condition for the performances of this work with the least unnecessary expenditure of force. These defects are not disease. The subject of them is not usually aware of their existence. The visual power may be good and the eyes themselves may feel no sense of strain, yet the nervous energy may be rapidly expended through their influence. There are some who by virtue of great reserve energy are able to contend with these difficulties for many years with little, if any, appreciable effect upon their general strength. How much more such persons might be able to accomplish were they free from such disadvantages, is a matter for conjecture. It is certain, however, that no worker who aims at the best results can afford needlessly to expend the power with which he must accomplish his work, and it is also certain that one who thus expends needlessly a considerable proportion of his working capital of nervous energy is so far at a disadvantage in the competitions of life.

